# Diversity of protein food sources, protein adequacy and amino acid profiles in Indonesia diets: Socio-Cultural Research in Protein Transition (SCRiPT)

**DOI:** 10.1017/jns.2022.82

**Published:** 2022-09-28

**Authors:** Helda Khusun, Pablo Monsivais, Roselynne Anggraini, Judhiastuty Februhartanty, Elise Mognard, Yasmine Alem, Mohd Ismail Noor, Norimah Karim, Cyrille Laporte, Jean-Pierre Poulain, Adam Drewnowski

**Affiliations:** 1 SEAMEO Regional Center for Food and Nutrition (RECFON) – Pusat Kajian Gizi Regional Universitas Indonesia, Jakarta, Indonesia; 2 Department of Nutrition and Exercise Physiology, Elson S. Floyd College of Medicine, Washington State University, Spokane, WA 99202, USA; 3 Taylor's Toulouse University Center and Faculty of Social Sciences & Leisure Management, Taylor's University, Selangor Darul Ehsan, Malaysia; 4 University of Toulouse Jean Jaurès, CERTOP UMR-CNRS 5044, Toulouse, France; 5 Centre for Community Health Studies (ReaCH), Faculty of Health Sciences, Universiti Kebangsaan Malaysia, Bandar Baru Bangi, Malaysia; 6 Center for Public Health Nutrition and Department of Epidemiology, University of Washington, Seattle, WA, USA

**Keywords:** Animal, Eating patterns, Ethnicity, Food choices, Plant, Protein transition, SES

## Abstract

The ongoing nutrition transition in lower- and middle-income countries (LMIC) in South East Asia may have a positive impact on protein nutrition. This study assessed the diversity of plant and animal protein food sources in relation to essential amino acid (EAA) adequacy in a population-based sample (*N* 1665) in Indonesia. Dietary intakes from in-person 24 h recalls provided data on energy and protein intakes (in g/d) from plants (grains, legumes), meat, poultry and fish, and eggs and dairy. Protein diversity scores were based on the number of protein food sources over 24 h. EAA scores were the ratio of amino acid intakes to recommended values. Protein diversity and EAA scores were then compared across multiple socio-demographic indices. Analysis of variance and *χ*^2^ tests were used to test for differences among groups. Energy intakes were 1678 kcal/d for men and 1435 kcal/d for women. Average protein intakes (and prevalence of inadequacy) were 59⋅4 g/d (41⋅7 %) for men and 51⋅5 g/d (51⋅1 %) for women. In regression analyses, higher protein diversity scores were associated with higher protein intakes, more animal protein and less plant protein and with higher EAA scores. Lower protein diversity scores were associated with lower intakes of lysine, leucine and valine relative to requirements, as well as with lower EAA, rural settings, less wealth and less modernisation. Greater diversity of animal protein food sources, observed among groups of higher socio-economic status, was linked to better amino acid adequacy and protein nutrition.

## Introduction

Lower- and middle-income countries (LMIC) in South East Asia are undergoing what has been described as a protein transition^(1)^. As country and household incomes rise, plant proteins from rice and legumes are being replaced by animal-source proteins from meat and poultry, eggs and dairy products^(1–3)^. An opposing protein transition is currently being promoted in high-income countries (HIC) whereby animal-source proteins are replaced by plant proteins^(4)^.

The main goal of the Socio-cultural Research in Protein Transition (SCRiPT) study, a population-based survey on dietary habits, was to track the key elements of the protein transition in Indonesia and Malaysia^(5,6)^. Indonesia is at an earlier stage of economic development as compared to Malaysia^(6–8)^ and is still classified among lower middle-income countries by the World Bank^(9)^. Current economic analyses suggest that Indonesia diets still resemble those of lower-income countries in se Asia, being dependent on a single starchy staple and characterised by still-low consumption of meat, eggs and dairy^(10)^. In the 2021 Global Hunger Index^(11)^, Indonesia ranks 73rd out of 116 countries with a moderate level of hunger, with the prevalence of childhood stunting estimated at 30⋅8 %^(12)^. The Global Hunger Index is a composite metric of undernourishment, stunting, wasting and under 5 mortality^(12)^.

The Food and Agriculture Organization of the United Nations (FAO) uses diversity metrics to assess diet quality, along with measures of dietary adequacy, moderation and balance^(13,14)^. Diversity of protein food sources is viewed as an important component of diet quality^(15)^, leading to adequate amino acid nutrition and an appropriate balance of essential amino acids. Even though Indonesia is undergoing the nutrition and protein transitions, diet quality is not equal across social and demographic groups^(16)^. The 2014 Indonesia Total Diet Study showed that most of the dietary protein came from plant-source as opposed to animal-source foods^(17)^. The SCRiPT study asked whether protein diversity varied across Indonesia socio-demographic groups^(5)^.

The present study used novel metrics to address protein adequacy, diversity and balance in the Indonesia diet. The first objective was to examine associations between the diversity of protein sources, protein adequacy and amino acid nutrition. The second objective was to assess how these metrics of protein diversity and quality varied across socio-demographic groups and with new composite metrics of urbanisation and modernisation. The present goal was to add to our understanding of protein nutrition in Indonesia, with implications for other LMICs in South East Asia.

## Materials and methods

### Participant selection and recruitment

Participants were drawn from West Sumatra, Jakarta, West Java, East Java, Bali and South Sulawesi. These provinces cover 48 % of the Indonesian population. Close to 80 % of the population resides in only fourteen provinces, most located on the Island of Java. Jakarta province was the most urbanised area, whereas West Java and East Java had the highest populations on Java Island. West Sumatra and Bali provided ethnic diversity and very distinct food cultures. East Indonesia was represented by South Sulawesi.

One urban district (most often the provincial capital) and one rural district were randomly selected within each province. For maximum power of analysis, number of participants from each province was approximately equal through over-sampling. Participant selection used multi-stage random sampling, using a cluster method. A cluster refers to village, i.e. the lower administrative level of district, consisting of approximately 400–550 households. The sampling procedure is illustrated by a diagram in Supplementary Figure S1.

The survey team visited selected households. Interviewers completed a household roster listing all household members and screened them for eligibility. A single eligible respondent from within the household was then selected at random. Written informed consent was obtained from all respondents and this study was conducted according to the guidelines laid down in the Declaration of Helsinki and all procedures involving research study participants were approved by the Human Ethical Committee of Universitas Indonesia (reference number 927/UN2.F1/ETIK/2017).

The sample size was powered to detect 10 % point differences in the prevalence of plant *v.* animal protein intake between any two groups. After accounting for clustered, multi-stage sample design, a minimum sample of 1548 adults was needed. For analysis purposes, the SCRiPT sample was weighted by population density, by urban rural location, age and sex. The weighting ensured that the SCRiPT sample was a national representative sample of the Indonesian population.

### Data collection methods and procedures

The SCRiPT project used a combination of quantitative dietary intake assessment along with in-person interviews. Dietary intakes data were collected using a structured questionnaire. The questionnaire and demographic surveys were pre-tested to ensure that the questions were appropriate, well understood and employed commonly used terms.

### Socio-demographic questionnaires

Sex was coded as male or female. Participants were first asked about their date of birth (day, month and year) and then about their age. Age groups were defined as 18–25 years, 26–35 years, 36–45 years and ≥46 years. Level of education was assessed using eight options and recoded as primary or lower school, lower secondary school, upper secondary school and college. The main occupation was captured using fifteen categories used in the Malaysian Food Barometer^(3)^ and was recoded as Professional (including managers, executives, medical doctors, teachers); White collar (including skilled technicians, clerical and service workers); Blue collar (including agriculture and fishery workers, construction work and cleaners); Students and Housewife. Ethnicity was recoded into eight categories reflecting a mix of ethnicity and the geographical location, consisting of (1) Minangkabau and other Sumatera/Malay ethnics, (2) Betawinese, (3) Sundanese, (4) Javanese, (5) Balinese, (6) All Sulawesi ethnics, (7) Madurese and (8) Others. Others consisted of fifty respondents into eleven identified ethnic groups (Other Eastern Indonesia ethnics and Chinese). These eight groups represent more than 74 % of the Indonesian population. Marital status was single (never married, divorced, separated, widowed) *v.* married or partnered.

### Wealth index (Indonesia) and modernisation scores

The composite ‘wealth index’, developed for this study, was based on housing conditions and ownership of household belongings. The thirteen input variables were: house wall material, floor material, type of toilet used, sources of electricity, sources of fuel for cooking, as well as ownership of car, bicycle, motorcycle, refrigerator, mobile phone, land line phone, television and radio. Scores were calculated based on principal component factors analysis with varimax rotation and were split into tertiles, with the lowest tertile (T1) representing the least wealth.

For urbanisation, all urban areas in Java Island provinces, Jakarta and Bali were categorised as urban, urban provinces in West Sumatra and South Sulawesi were categorised as suburban and other areas were categorised as rural. The Central Bureau of Statistics^(18)^ defines urbanisation on the basis of population density, availability of urban facilities, percent households with phones and using electricity.

The composite modernisation index was based on wealth index tertiles, urbanisation and number of children. For wealth index tertile, the point scores were: lowest tertile = 10, medium tertile = 30 and highest tertile = 50. For urbanisation, point scores were urban = 50; suburban = 30 and rural = 10. For the number of children, the point scores were: 0–2 children = 50; 3–4 children = 30 and 5 or more children = 0.

### Dietary intakes from 24 h recall

Dietary intakes data were collected by face-to-face interview using a structured questionnaire to probe for all foods consumed over a 24 h period. Multiple pass method was used for data collection. Initially, respondents were asked to list all the foods and drinks they consumed the day before during the waking hours. For each food, respondents estimated the amounts eaten using a photographic atlas of portion sizes. A single 24 h recall was used, with data collected on weekdays.

Energy and nutrient intakes were calculated using a customised version of the NutriSurvey for Windows 2007. First, the latest 2017 version of the Indonesian Food Composition Table^(19)^ was manually entered into the software. The Indonesian FCT consists of 1128 foods (including cooked foods/ menu), categorised into 13 food groups and provides data for energy, water (moisture content) and 19 nutrients. The present nutrients of interest were energy (kcal) and protein (g). Data on grams of protein from local foods or regional mixed dishes in Indonesia were not available.

### Protein diversity score

We developed a protein diversity score based on protein intakes in g/d from each food source from 24 h dietary recalls^(5)^. The protein food sources were coded as cereals, legumes, eggs, dairy, beef, pork, poultry and fish. Estimated gram daily intakes from each protein food source were summed for each participant. Intakes >0 g/d were scored ‘1’ for intake of that source of protein. The protein diversity score had a theoretical range of 1 to 8 and was stratified into four groups for analysis.

### Protein adequacy and amino acid ratios

Protein adequacy of individuals was calculated as the ratio between estimated intake of total dietary protein and adequate intake, from the formula: Body Weight (kg) × 0⋅83 g, based on WHO, FAO & UNU^(20)^. A dichotomous variable for protein adequacy was derived for each individual, with the ratio of estimated intake to requirements for adequacy of one or greater classified as adequate (ratio ≥1) else inadequate. An additional threshold was examined, with an adequate ratio defined as ≥0⋅7. Mean percent adequacy score for essential amino acids was computed for each survey respondent, based on minimum protein requirements according to the 2007 FAO/WHO/UNU report on protein and amino acid requirements. The adequacy of intake for each of the EAAs (histidine, isoleucine, leucine, lysine, threonine, tryptophane, valine, methionine + cysteine and phenylalanine + tyrosine) was computed based on the ratio between estimated intake and the requirements given each individual's protein adequacy requirements^(20)^. The mean adequacy ratio (MAR) for all EAAs was computed as the arithmetic mean of nine ratios. The MAR was dichotomised with mean adequacy defined as a ratio of 1 or greater.

### Plan of data analysis

The survey sample had more young urban males (18–24 years), more urban females (30–39 years) and more rural females (ages 20–39 years) than the national averages. The sample was then weighted by population density, by urban rural location, age and sex to make it representative of the Indonesian population. Univariate tests of differences across socio-demographic groups were tested using one-way ANOVA. Associations between intake of protein and protein diversity and socio-demographic variables (e.g. age, gender, wealth, modernisation, urbanisation) and cultural variables (ethnicity, area) were assessed based on one-way ANOVA and *χ*^2^ tests. Associations between protein diversity and adequacy of protein and essential amino acid (EAA) intakes were assessed with logistic regression models. Analyses were conducted using SPSS (v. 28) and SAS statistical programs.

## Results

### Energy intakes and protein nutrition

A total of 1727 Indonesian adults were surveyed, of whom 62 were excluded from analyses because of missing data. Table 1 shows estimated energy intakes (kcal/d), protein intakes (g/d) and protein diversity and protein adequacy scores, all based on 24 h dietary recalls, across selected socio-demographic variables in the full, weighted sample (*n* 1665). The sample was equally split between men and women, by age strata and across modernisation indices. Most participants had children (76 %) and most lived in urban areas (67 %). About 27 % had only primary education and 44 % finished upper secondary school.

**Table 1.** Intake of dietary energy, protein and essential amino acids, based on 24 h recalls, by socio-demographic characteristics

Socio-demographics			Energy kcal/d	Protein g/d	Protein inadequacy^a^	Protein diversity^b^	EAA MAR <1^c^
	Count	%	Mean ± sem	Mean ± sem	% Inadequate	Mean ± sem	%
**All**	1665	100	1559 ± 15	55⋅6 ± 0⋅7	46⋅2	4⋅81 ± 0⋅03	25⋅2
**Gender**							
Male	858	51⋅5	1678 ± 21	59⋅4 ± 0⋅9	41⋅7	4⋅78 ± 0⋅04	23⋅1
Female	807	48⋅5	1435 ± 21	51⋅5 ± 0⋅9	51⋅1	4⋅83 ± 0⋅04	27⋅5
*P-Value*^d^			<0⋅001	<0⋅001	<0⋅001	0⋅391	0⋅037
**Age groups (years)**							
18–25	354	21⋅2	1642 ± 34	59⋅4 ± 1⋅6	37⋅0	4⋅81 ± 0⋅06	19⋅8
26–35	476	28⋅6	1648 ± 30	57⋅5 ± 1⋅3	44⋅1	5⋅01 ± 0⋅06	25⋅4
36–45	337	20⋅3	1556 ± 34	56⋅1 ± 1⋅5	50⋅0	4⋅85 ± 0⋅06	25⋅2
≥46	498	29⋅9	1419 ± 24	50⋅6 ± 1⋅1	52⋅2	4⋅58 ± 0⋅05	28⋅7
*P-Value*^d^			<0⋅001	<0⋅001	<0⋅001	<0⋅001	0⋅032
**Number of children**							
No children	402	24⋅1	1595 ± 30	56⋅6 ± 1⋅4	40⋅5	4⋅83 ± 0⋅06	23⋅6
1–2 children	775	46⋅5	1611 ± 23	57⋅3 ± 1⋅0	45⋅7	4⋅90 ± 0⋅04	24⋅6
3–4 children	356	21⋅4	1449 ± 30	51⋅6 ± 1⋅3	52⋅8	4⋅73 ± 0⋅06	27⋅0
5 or more children	132	7⋅9	1445 ± 49	52⋅9 ± 2⋅4	48⋅9	4⋅42 ± 0⋅09	28⋅2
*P-Value*^d^			<0⋅001	0⋅006	0⋅008	<0⋅001	0⋅597
**Wealth index**							
Tertile 1	554	33⋅3	1585 ± 30	55⋅4 ± 1⋅3	46⋅8	4⋅49 ± 0⋅05	26⋅9
Tertile 2	559	33⋅6	1547 ± 25	55⋅2 ± 1⋅2	45⋅8	4⋅91 ± 0⋅05	25⋅8
Tertile 3	553	33⋅2	1547 ± 25	56⋅1 ± 1⋅1	46⋅2	5⋅03 ± 0⋅05	22⋅8
*P-Value*^d^			0⋅490	0⋅834	0⋅103	<0⋅001	0⋅268
**Urbanisation**							
Urban	1124	67⋅5	1542 ± 18	55⋅0 ± 0⋅8	48⋅9	4⋅95 ± 0⋅04	26⋅9
Rural	541	32⋅5	1595 ± 28	56⋅8 ± 1⋅3	40⋅7	4⋅52 ± 0⋅05	21⋅6
*P-Value*^d^			0⋅102	0⋅205	0⋅002	<0⋅001	0⋅020
**Modernisation**							
Low	380	22⋅8	1547 ± 34	54⋅3 ± 1⋅4	44⋅2	4⋅42 ± 0⋅05	24⋅2
Low middle	433	26⋅0	1604 ± 32	58⋅5 ± 1⋅5	43⋅9	4⋅67 ± 0⋅06	25⋅4
High middle	462	27⋅7	1499 ± 28	52⋅0 ± 1⋅2	52⋅4	4⋅97 ± 0⋅06	29⋅0
High	390	23⋅4	1593 ± 29	57⋅8 ± 1⋅3	43⋅6	5⋅15 ± 0⋅06	21⋅5
*P-Value*^d^			0⋅050	<0⋅001	0⋅021	<0⋅001	0⋅089
**Education**							
Primary or lower	448	26⋅9	1505 ± 31	53⋅6 ± 1⋅4	45⋅9	4⋅38 ± 0⋅05	29⋅2
Lower secondary school	302	18⋅1	1562 ± 35	53⋅3 ± 1⋅4	50⋅2	4⋅86 ± 0⋅06	26⋅1
Upper secondary school	727	43⋅7	1583 ± 23	57⋅5 ± 1	44⋅7	5⋅00 ± 0⋅04	23⋅0
College/University	188	11⋅3	1592 ± 43	56⋅6 ± 1⋅9	46⋅6	5⋅07 ± 0⋅08	22⋅9
*P-Value*^d^			0⋅172	0⋅043	0⋅459	<0⋅001	0⋅090

a Protein inadequacy defined as individual with ratio of estimated intake to requirements of < 1, with requirements based on 0⋅83 g protein per kg body weight, from reference 20.

b Protein diversity based on intakes of eight categories of dietary protein, from single 24 h recalls.

c Essential amino acid (EAA) mean adequacy ratio (MAR) <1 defined as individuals with mean ratio of estimated EAA intake to requirements of <1.

d All *P* values are for univariate ANOVA except for percent-based comparisons, which are from *χ*^2^ analysis.

Mean energy intake was 1559 kcal/d; 1678 kcal/d for men and 1435 kcal/d for women. Energy intake was higher for men than for women (*P* < 0⋅001). Energy intakes varied significantly among age groups and households with different numbers of children. No significant differences in total energy intakes were observed by education, wealth, urbanisation or modernisation variables.

Mean protein intake estimated from 24 h recalls was 55⋅6 g/d. As with energy intake, protein intake was higher for men (59⋅4 g/d) than for women (51⋅5 g/d) and varied significantly with age group, children in the household, educational attainment and modernisation, but not with wealth index or urbanisation.

**Fig. 1.**
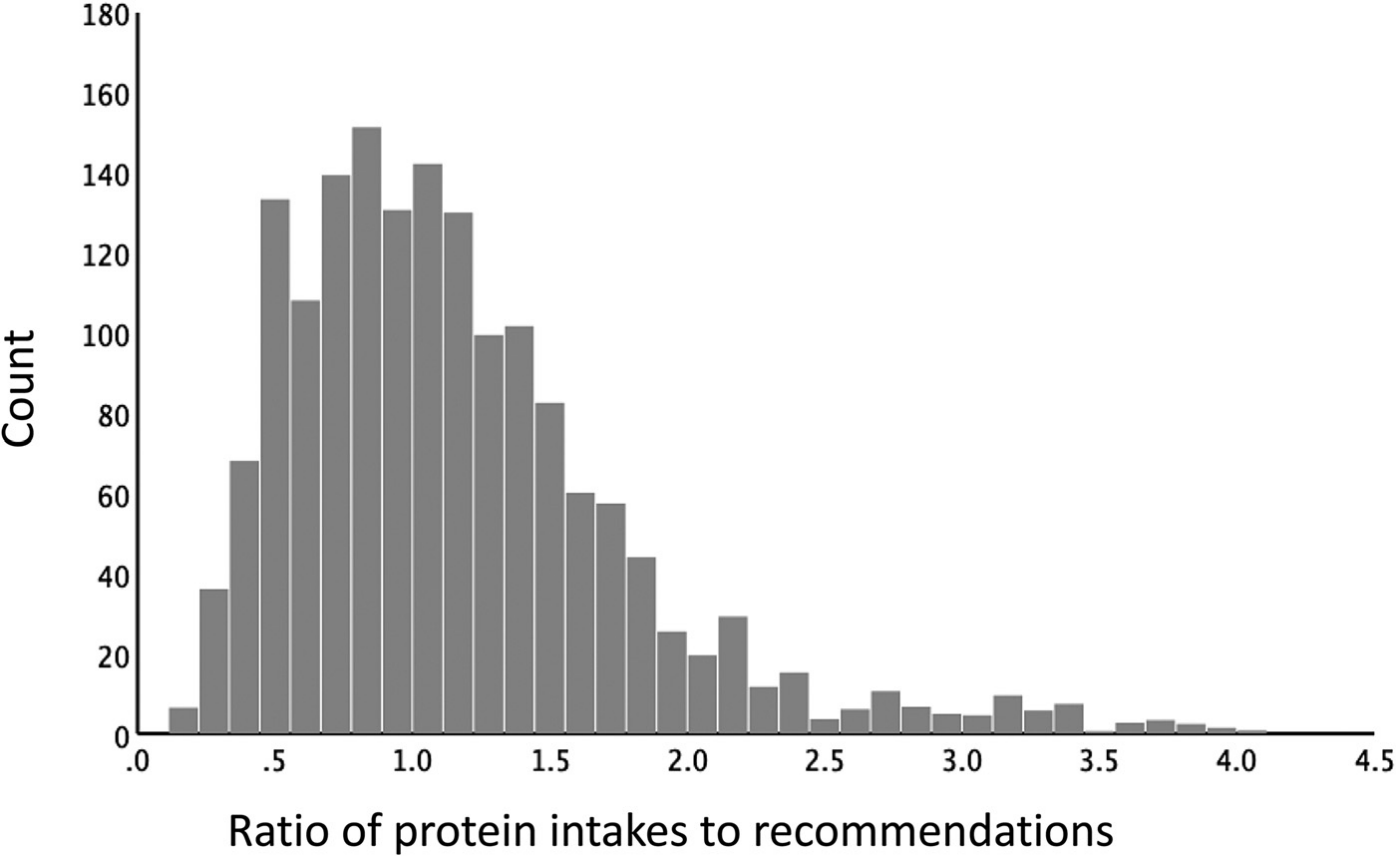
Histogram of the ratio of protein intake (from single 24 h recall) to adequate intakes based on body weight^(20)^. Analytic weighted sample of Indonesian adults, *n* 1665.

Overall, approximately 46 % of the sample was classified as having inadequate protein intake (ratio of estimated intake of protein to requirements <1) and there was wide variation in the adequacy of intakes (Fig. 1). Prevalence of protein inadequacy was higher for women, older adults and urban respondents. Protein inadequacy followed a less-consistent pattern for modernisation, but the prevalence of protein inadequacy varied by level of modernisation.

Mean diversity of protein food sources was 4⋅81 points out of 8 points. Protein diversity varied significantly by age group, number of children in the household and across all three indices of socio-economic status: educational attainment, wealth index and modernisation. Generally, protein diversity co-varied with protein adequacy but there were exceptions: for example, rural areas showed a lower prevalence of protein inadequacy but also lower protein diversity compared to urban areas. Overall, approximately 25 % of the sample showed a MAR for EAA intake of <1 and there were some significant variations among groups including by sex, age and urbanisation.

### Dietary protein diversity and amino acid adequacy ratios

**Fig. 2.**
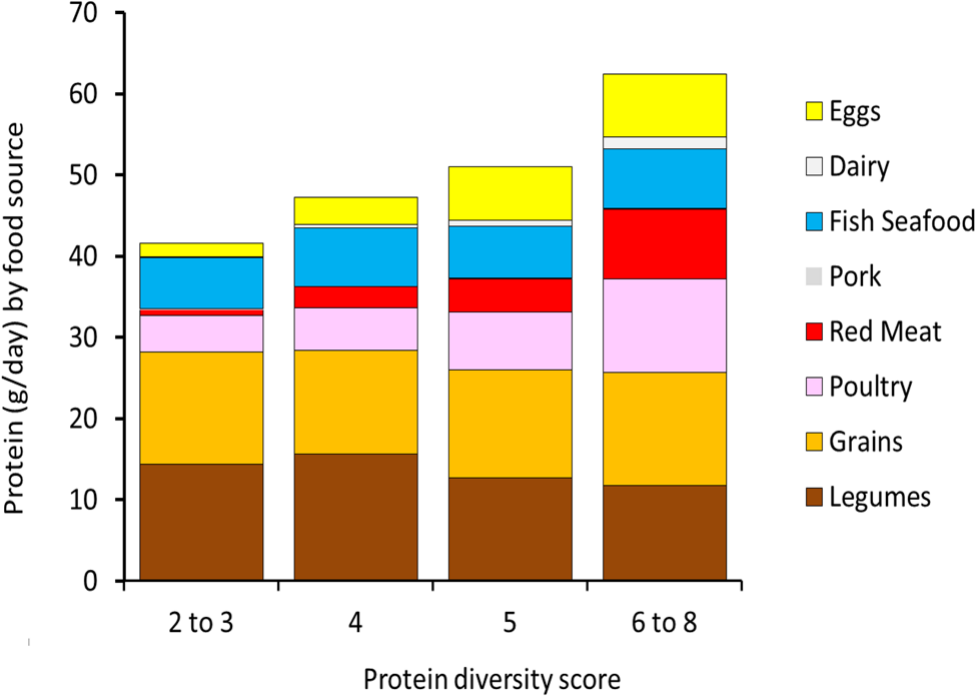
Protein intakes (in g/d) from food sources by protein diversity score.

Fig. 2 shows that higher protein diversity scores were associated with lower intakes of grains and legumes. By contrast, higher protein diversity scores were associated with higher intakes of animal protein, particularly poultry (chicken), beef and eggs. Intakes of fish protein were constant across protein diversity strata and the consumption of dairy was negligible.

**Fig. 3.**
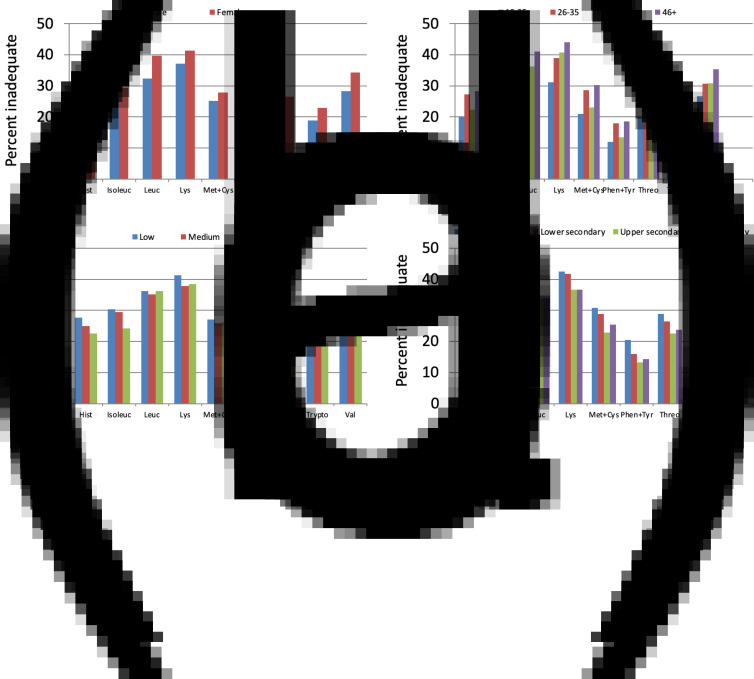
Prevalence of inadequacy for essential amino acids by sex (a), age group (b), household wealth tertile (c) and educational attainment (d). Hist, histidine; isoleuc, isoleucine; leuc, leucine; Lys, lysine; Met + Cys, methionine + cysteine; Phen + Tyr, phenylalanine + tyrosine; Threo, threonine; Trypto, tryptophan; Val, valine.

Table 2 shows the relation between protein diversity scores and total protein intakes (in g/d) from multiple protein food sources, both animal and plant. First, the amount of animal protein consumed varied significantly by protein diversity scores, with the greatest levels of animal protein among the highest diversity score group; by contrast, the amounts of plant proteins from cereals and legumes remained constant. Second, protein adequacy significantly varied across the level of protein diversity, with those with the lower dietary protein diversity having higher prevalence of protein inadequacy and those with the lowest protein diversity having 3⋅88 greater odds of protein inadequacy compared to those with the highest protein diversity (*P* < 0⋅001). Third, the prevalence of inadequate EAA intake ranged from 4 to 35⋅6 % and varied significantly across levels of protein diversity. Those with the lowest protein diversity have 6⋅56 greater odds of EAA inadequacy (*P* < 0⋅001). Although the adequacy of intakes for all EAAs was greatest among those with the highest protein diversity, the strongest gradients were shown by leucine, valine and lysine. These three EAAs also showed the highest overall shortfalls in the population. Fig. 3 shows that the prevalence of inadequate intake for the nine EAAs ranged from less than 12 % to more than 40 % and EAA inadequacy varied by sex, age, wealth and educational attainment.

**Table 2.** Intake of dietary energy, protein and essential amino acids, based on 24 h recalls, by level of dietary protein diversity

	Protein diversity strata^a^					
	2–3	4	5	6–8	Total	*P*-value^b^
*N*=	226	431	531	477	1665	
Energy 1 d intake (kcal)	1296 ± 43	1406 ± 29	1570 ± 24	1811 ± 27	1559 ± 15	<0⋅001
Protein 1 d intake (g)	44⋅8 ± 1⋅8	50⋅6 ± 1⋅4	54⋅7 ± 1⋅1	66⋅1 ± 1⋅2	55⋅6 ± 0⋅7	<0⋅001
Protein from animal sources^c^, g/d	13⋅5 ± 1⋅2	18⋅8 ± 0⋅8	25⋅0 ± 0⋅8	36⋅7 ± 1⋅0	25⋅1 ± 0⋅5	<0⋅001
Total protein from plant sources^d^, g/d	28⋅2 ± 1⋅8	28⋅4 ± 1⋅2	26⋅0 ± 0⋅8	25⋅7 ± 0⋅7	26⋅8 ± 0⋅5	0⋅116
Ratio of intakes to requirements						
*Total protein*	0⋅95 ± 0⋅04	1⋅05 ± 0⋅03	1⋅13 ± 0⋅02	1⋅37 ± 0⋅03	1⋅16 ± 0⋅02	<0⋅001
*Histidine*	1⋅38 ± 0⋅06	1⋅60 ± 0⋅05	1⋅78 ± 0⋅04	2⋅23 ± 0⋅05	1⋅81 ± 0⋅03	<0⋅001
*Isoleucine*	1⋅23 ± 0⋅05	1⋅44 ± 0⋅05	1⋅59 ± 0⋅04	1⋅95 ± 0⋅04	1⋅61 ± 0⋅02	<0⋅001
*Leucine*	1⋅07 ± 0⋅04	1⋅25 ± 0⋅04	1⋅38 ± 0⋅03	1⋅68 ± 0⋅04	1⋅39 ± 0⋅02	<0⋅001
*Lysine*	1⋅04 ± 0⋅05	1⋅22 ± 0⋅04	1⋅36 ± 0⋅03	1⋅77 ± 0⋅05	1⋅40 ± 0⋅02	<0⋅001
*Methionine + Cysteine*	1⋅21 ± 0⋅05	1⋅40 ± 0⋅04	1⋅64 ± 0⋅04	2⋅04 ± 0⋅05	1⋅64 ± 0⋅02	<0⋅001
*Phenylalanine + Tyrosine*	1⋅65 ± 0⋅07	1⋅94 ± 0⋅06	2⋅17 ± 0⋅05	2⋅65 ± 0⋅06	2⋅18 ± 0⋅03	<0⋅001
*Threonine*	1⋅37 ± 0⋅06	1⋅59 ± 0⋅05	1⋅75 ± 0⋅04	2⋅18 ± 0⋅05	1⋅78 ± 0⋅03	<0⋅001
*Tryptophan*	1⋅51 ± 0⋅06	1⋅73 ± 0⋅06	1⋅89 ± 0⋅04	2⋅25 ± 0⋅05	1⋅90 ± 0⋅03	<0⋅001
*Valine*	1⋅09 ± 0⋅05	1⋅29 ± 0⋅04	1⋅44 ± 0⋅03	1⋅73 ± 0⋅04	1⋅43 ± 0⋅02	<0⋅001
Mean adequacy ratio (MAR)	1⋅28 ± 0⋅05	1⋅50 ± 0⋅05	1⋅67 ± 0⋅04	2⋅05 ± 0⋅05	1⋅68 ± 0⋅02	<0⋅001
Percent with MAR <1 for essential amino acids	43⋅8	34⋅1	23⋅2	10⋅5	25⋅2	<0⋅001
Odds ratio^e^ (95 % CI) of MAR <1	6⋅56 (4⋅43, 9⋅72)	4⋅39 (3⋅08, 6⋅25)	2⋅56 (1⋅79, 3⋅64)	reference		<0⋅001
Percent with inadequate total protein intake^f^	64⋅3	53⋅9	45⋅6	31⋅6	46⋅2	<0⋅001
Odds ratio^e^ (95 % CI) of inadequate total protein intake	3⋅88 (2⋅78, 5⋅41)	2⋅54 (1⋅94, 3⋅33)	1⋅82 (1⋅41, 2⋅36)	reference		<0⋅001

a Protein diversity strata based on intakes of eight categories of dietary protein, from single 24 h recalls.

b All *P* values are for univariate ANOVA except for percent-based comparisons, which are from *χ*^2^ analysis.

c Animal sources of protein included poultry, meat, fish, eggs and dairy.

d Plant protein sources included grains and legumes.

e Odds ratios and 95 % confidence intervals estimated from bivariate logistic regressions.

f Protein inadequacy defined as individual with ratio of estimated intake to requirements of <1, with requirements based on 0⋅83 g protein per kg body weight, from reference 20.

## Discussion

The present analyses provide new insights into protein intakes and the diversity of dietary protein sources in Indonesia.. First, energy intakes from 24 h recalls were comparable to those from a 2018 survey of adults in urban and rural West Java^(19)^, which reported energy intake of 1698 kcal/d for men and 1411 kcal/d for women. In the present population-based sample, energy intakes varied by age and gender, occupation, ethnicity and the number of children. Comparable trends in energy intakes by age, marital status, occupation and wealth index had been observed before^(19,21)^.

Mean protein intakes were 55⋅4 g/d, below the 65 g/d value recommended by the Ministry of Health. Estimated mean protein intakes in g/d also varied by age, ethnicity, province and the number of children. The protein diversity score, defined in terms of the number of protein food sources varied significantly in relation to multiple indicators of socio-economic status.

Higher protein diversity scores were given to diets with that contained more total protein and more animal protein from chicken, red meat and eggs. The consumption of milk and dairy products was generally low. Diets that were high in starchy staples (rice) received lower protein diversity scores. The finding that diets with more animal foods and lower consumption of starchy staples were associated with higher socio-economic status was consistent with Bennett's law^(22)^. Bennett's law of economics states that as incomes rise, people (and nations) eat fewer starchy staples and derive more animal protein from poultry, red meat and eggs. In other words, consumer demand for animal protein foods is driven by economics.

Using food balance sheets, rather than consumption data, the FAO has estimated the protein composition of the Indonesia diet at 81 % from plant proteins, mostly rice and legumes (soja/*tempeh*) and only 19 % from animal protein, of which 57 % was from fish^(23)^. Rising meat consumption in se Asia has followed economic development^(22)^, though some regional preferences have been observed^(24)^. Consistent with our findings, Indonesia and Malaysia prefer poultry, while some countries and regions prefer pork^(21)^. Beef and dairy production and consumption are quite modest throughout the region^(1)^. Based on US Department of Agriculture projections^(25)^, the production and consumption of pork and poultry in se Asia will grow over the next decade, outpacing beef and fish.

The Indonesia diet is still built around rice and legumes. In the Indonesia National Total Diet Study^(17)^, the reported diet composition by food group was cereals (mostly rice) 257⋅7 g/d, legumes 56⋅7 g/d, fish 78⋅4 g/d, meat and poultry 42⋅8 g/d, egg 19⋅7 g/d and milk 5 g/d. By contrast, there are very limited data on the relative contribution of protein from each food source at the national level or across socio-economic groups. The recommended protein daily values for Indonesian adults are 65 g/d for men and 60 g/d for women, corresponding to 20–25 % of energy intake from protein^(17)^. Those values are higher than the normal standard of 50 g protein per day.

As incomes increase, Indonesia will most likely follow the path of Malaysia. The SCRiPT studies, conducted in parallel in Indonesia and Malaysia offer a potential glimpse into the future. Malaysia is farther along the scale of economic development compared to Indonesia. In Malaysia, animal proteins accounted for about 66 % of total protein intakes^(6)^, levels comparable to those observed in HIC such as France^(24)^ and the US^(26)^. Diets with higher proportions of animal protein in Malaysia were more common among younger urban dwellers with higher education and incomes^(5)^.

It is worth noting that fish consumption represents a special case. Fish has been the traditional source of high-quality protein, especially for the rural poor. Intakes of fish protein did not vary with the diversity score. Fish consumption may explain why less varied diets in Indonesia were adequate in protein^(21)^. However, based on recent reports, fish consumption in both Indonesia and Malaysia has become associated with rural (island) regions, larger families and lower socio-economic status^(5, 21)^.

The present data offer new insights into the ongoing nutrition transition across low- and middle-income countries in se Asia^(10,27–29)^. First, the choice of animal proteins can be context-dependent. In Indonesia, the favoured sources were poultry, eggs and dairy, with less frequent beef and very little or no pork. There were also distinctions to be made within the animal protein category. Diets with a higher proportion of meat, poultry and eggs were associated with higher socio-economic status, and younger age groups with higher education and incomes.

The present assessments of protein and amino acid nutrition relative to requirements point to a link between the diversity of protein food sources and the adequacy of intakes at the population level^(20,30,31)^. Typically, these measures standardise the intake of protein and amino acid intakes on a per-person basis, depending on age^(20)^ or energy requirements^(30,32)^. In this study, we used estimated protein requirements from the WHO/FAO, finding an overall prevalence of protein inadequacy of approximately 30 % and significant variation among some socio-demographic populations. Further, the prevalence of protein inadequacy was strongly associated with the diversity of protein sources. Those with the lowest protein diversity had a prevalence of protein inadequacy of 49 % and consumed levels of lysine, leucine and valine that were close to minimal requirements. There was substantial variation among demographic groups in the overall adequacy of EAA intakes. Among individual essential amino acids, leucine, lysine and valine showed the strongest variation with dietary protein diversity.

One limitation of SCRiPT Indonesia was that most participants came from the most densely populated provinces. Even though the present results can be generalised to the Indonesian population, there are populations at risk that merit further study. Second, dietary intakes were limited to a single 24 h recall, which may have led to recall error and bias as well as underestimated intakes of foods and beverages that are consumed infrequently^(33)^. It should be noted that present mean energy intakes were below those in the 2014 Indonesia Total Diet Study, which reported a mean of 1805 kcal/d (men 1998 kcal/d; women 1607 kcal/d)^(17)^ and estimated mean protein intakes at 65 g/d (men 70⋅5 g/d; women 59⋅2 g/d) for both urban and rural location^(17)^. Another study reported higher energy intakes among urban dwellers (men 2165 kcal/d; women 1817 kcal/d relative to the present results^(8,34)^. Finally, the diversity score was based on the count of protein food sources and not the amount and was at best a proxy measure of protein quality. This method to assess diversity would have over-counted foods or beverages consumed in small amounts, since any intake (>0 g per d) would have contributed to the diversity score. However, the score was linked to protein intakes and amino acid adequacy, which would be expected if the score was indicative of the diversity of the protein sources in the diet.

## Conclusions

Lowest protein diversity scores in the present sample were associated with minimally adequate protein intakes. Low protein diversity was reflected in lower intakes of essential amino acids lysine, leucine and valine. More diverse diets that include animal-based foods may help ensure the adequacy of protein and amino acid nutrition across the LMIC. Future studies may include protein quality screeners to assess protein nutrition among Indonesia population subgroups.

## Supplementary material

For supplementary material accompanying this paper visit https://doi.org/10.1017/jns.2022.82. click here to view supplementary material
