# Supplementary material for: Diversity of protein food sources, protein adequacy and amino acid profiles in Indonesia diets: Socio-Cultural Research in Protein Transition (SCRiPT)
Source: J Nutr Sci. 2022 Sep 28;11:e84. doi: 10.1017/jns.2022.82 (PMC9554421; doi:10.1017/jns.2022.82)
Supplement: Supplementary file 1 [file S2048679022000829sup001.docx]

| **Supplementary Figure 1:** Flow of the sampling procedure**.** Participant selection was based on multi-stage random sampling, using cluster method, proportionate-to-population size (PPS). HH, household; RT, Rukun Tetangga, Indonesia’s network of neighborhood associations and the lowest administrative division of Indonesia. |
| --- |
| List of village names and number of population in each Village at the 20 cities  PPS method  List of selected villages  ss  Random selection method  List of selected hamlets in each village (4-5 hamlet in each village)  Random selection method  List of selected RT in each hamlet (1 RT in each hamlet)  Randomly select HH in each RT  (List of HH will be obtained from Head of selected RT)  List of selected household in each RT |

| **Supplementary Table 1.** Adequacy of protein intake by sociodemographic characteristics | | | | |
| --- | --- | --- | --- | --- |
| **Socio-demographics** |  |  | **Protein inadequacy^1^** |  |
|  | **Count** | **%** | % Inadequate |  |
| **All** | 1665 | 100 | 23.1 |  |
| **Gender** |  |  |  |  |
| Male | 858 | 51.5 | 19.9 |  |
| Female | 807 | 48.5 | 26.5 |  |
| *P-Value^2^* |  |  | 0.002 |  |
| **Age groups (years)** |  |  |  |  |
| 18-25 | 354 | 21.2 | 18.6 |  |
| 26-35 | 476 | 28.6 | 21.8 |  |
| 36-45 | 337 | 20.3 | 24.6 |  |
| >46 | 498 | 29.9 | 26.5 |  |
| *P-Value^2^* |  |  | 0.045 |  |
| **Number of Children** |  |  |  |  |
| No children | 402 | 24.1 | 23.4 |  |
| 1-2 children | 775 | 46.5 | 21.7 |  |
| 3-4 children | 356 | 21.4 | 24.6 |  |
| 5 or more children | 132 | 7.9 | 26.5 |  |
| *P-Value^2^* |  |  | 0.522 |  |
| **Wealth index** |  |  |  |  |
| Tertile 1 | 554 | 33.3 | 24.2 |  |
| Tertile 2 | 559 | 33.6 | 23.6 |  |
| Tertile 3 | 553 | 33.2 | 21.5 |  |
| *P-Value^2^* |  |  | 0.541 |  |
| **Urbanization** |  |  |  |  |
| Urban | 1124 | 67.5 | 24.1 |  |
| Rural | 541 | 32.5 | 21.1 |  |
| *P-Value^2^* |  |  | 0.168 |  |
| Table continued on next page | | | | |

| **Supplementary Table 1 (Cont.)** | | | |  |
| --- | --- | --- | --- | --- |
| **Socio-demographics** |  |  | **Protein inadequacy^1^** |  |
|  |  | **Count** | **%** |  |
| **Modernization** |  |  |  |  |
| Low | 380 | 22.8 | 23.7 |  |
| Low middle | 433 | 26.0 | 19.4 |  |
| High middle | 462 | 27.7 | 28.9 |  |
| High | 390 | 23.4 | 19.7 |  |
| *P-Value^2^* |  |  | 0.002 |  |
| **Education** |  |  |  |  |
| Primary or lower | 448 | 26.9 | 26.1 |  |
| Lower secondary school | 302 | 18.1 | 23.5 |  |
| Upper secondary school | 727 | 43.7 | 21.3 |  |
| College ‎/ University | 188 | 11.3 | 22.3 |  |
| *P-Value^2^* |  |  | 0.299 |  |
| ^1^ Adequacy defined as individual with ratio of estimated intake to requirements of < 0.7, with requirements based on 0.83 g protein per kg body weight, from reference 21; ^2^ P-values from Chi-squared tests | | | |  |

| **Supplementary Table 2.** Adequacy of protein intake based on 24h recalls, by level of dietary protein diversity | | | | | | | |
| --- | --- | --- | --- | --- | --- | --- | --- |
|  | **Protein diversity strata^1^** | | | | | | |
|  | **2—3** | **4** | **5** | **6—8** | **Total** | **p-value^2^** |  |
| N= | 226 | 431 | 531 | 477 | 1665 |  |  |
|  |  |  |  |  |  |  |  |
| Percent with inadequate total protein intake ^3^ | 43.8 | 32.9 | 20.8 | 7.1 | 23.1 | <0.001 |  |
| Odds ratio ^4^ (95% CI) of inadequate total protein intake | 10.05  (6.50 15.56) | 6.37  (4.26, 9.53) | 3.41  (2.27, 5.13) | reference |  | <0.001 |  |
| ^1^ Protein diversity strata based on intakes of eight categories of dietary protein, from single 24h recalls; ^2^ P values for percent inadequate from Chi-square analysis; ^3^ Protein inadequacy defined as individual with ratio of estimated intake to requirements of < 0.7, with requirements based on 0.83 g protein per kg body weight, from reference 21; ^4^ Odds ratios and 95% confidence intervals estimated from bivariate logistic regressions. | | | | | | |  |
